# Adherence of critical care nurses to endotracheal suctioning guidelines: a cross-sectional study

**DOI:** 10.1186/s12912-022-01092-w

**Published:** 2022-11-14

**Authors:** Sameer A. Alkubati, Khaled M. Al-Sayaghi, Gamil G. Alrubaiee, Mokhtar Abdu Hamid, Khalil A Saleh, Talal Al-Qalah, Ahmad K. Al-Sadi

**Affiliations:** 1 Department of Medical Surgical, College of Nursing, University of Hail, Hail, Saudi Arabia; 2 Department of Nursing, Faculty of Medicine and Health Sciences, Hodeida University, Hodeida, Yemen; 3 Department of Medical Surgical Nursing, College of Nursing, Taibah University, Al-Madinah Al-Munawarah, 42353 Saudi Arabia; 4 Nursing Division, Faculty of Medicine and Health Sciences, Sana’a University, Sana’a, 1247 Yemen; 5 Department of Community Health, College of Nursing, Faculty of Medical Sciences, Al-Razi University, Sanaa, Yemen; 6 Faculty of Nursing, Taiz University, Taiz, Yemen

**Keywords:** Suction, Endotracheal intubation, Practice, Guideline adherence, Critical care nursing, Intensive care units

## Abstract

**Background:**

Endotracheal suctioning (ETS) is one of the most common invasive procedures performed by critical care nurses (CCNs) to remove accumulated pulmonary secretions, ensure airway patency for adequate ventilation and oxygenation as well as prevent atelectasis in intubated patients.

**Objectives:**

To assess the practice of CCNs in intensive care units (ICUs) before, during, and after performing the ETS procedure and identify factors affecting their practice.

**Methods:**

A cross-sectional and non-participant observational design was conducted in the ICUs of four hospitals in Hodeida city, Yemen. The data were collected using a 25-item observational checklist in the period from May to August 2019.

**Results:**

More than half (55%) of CCNs scored undesirable (< 50%) regarding their adherence to ETS practice guidelines while the rest scored moderate (50–75%), with none of showing desirable adherence (> 70%) to the guidelines. There was no significant association between gender, age, education level, or length of experience of CCNs in the ICUs and their practice during performance ETS procedures. However, training (*p* = 0.010) and receiving information about ETS (*p* = 0.028) significantly improved the CCNs’ practice.

**Conclusion:**

Most CCNs at the ICUs of Hodeida hospitals do not adhere to evidence-based practice guidelines when performing ETS procedures, possibly resulting in numerous adverse effects and complications for patients. CCNs receiving information and training show better ETS practice than do their counterparts. Therefore, it is necessary to provide the nursing staff with clear guidelines, continuous education and monitoring to improve their practices.

## Introduction

Endotracheal tubes (ETTs) may suppress ciliary movement and cough reflex within hours after endotracheal intubation in critically ill patients, resulting in the accumulation of biofilm secretions and constriction and occlusion of airways [1–3]. Consequently, such patients may experience some health problems such as airway obstruction, hypoxemia, hypercapnia, alveolar collapse, and infection [4]. Endotracheal suctioning (ETS) is one of the most common invasive procedures performed by critical care nurses (CCNs) to remove accumulated pulmonary secretions [4–6], and hence ensure airway patency for adequate ventilation and oxygenation as well as prevent atelectasis [7, 8].

ETS is a procedure that involves catheter insertion through the ETT to remove the airway secretions by applying a negative pressure [9, 10]. If ETS is not performed according to evidence-based practice, it may lead to many complications such as tracheobronchial trauma and bleeding, ventilator-associated pneumonia, ulceration, atelectasis, hypoxemia, cardiovascular instability and elevated intracranial pressure [4, 5, 9]. Studies have shown that ETS complications can be prevented by adhering to evidence-based guidelines when performing the procedure [11, 12], reducing the cost of health care by shortening the length of hospital stay [11]. Accordingly, CCNs play an important role in minimizing and preventing potential problems and complications by providing safe and effective up-to-date ETS practices to ensure the delivery and appropriate quality of care [9, 13].

Several evidence-based practice recommendations have been developed to improve the clinical practice of CCNs when performing ETS [14–16]. However, there is a gap between these recommendations and the actual practice of CCNs. Unexpected findings were found in European countries, where studies revealed poor practices and non-adherence of CCNs to the recommendations for suctioning during ETS [8, 17]. On the other hand, a few studies have been published on this issue in developing countries [9] and, to our knowledge, no studies have been conducted in Yemen to assess the CCNs’ practice regarding ETS. The findings of this study can raise the awareness of CCNs and medical administrators about planning and implementing ETS to improve its practice**.** Therefore, the present study aimed to assess the CCNs’ practice before, during, and after performing the ETS procedure and identify the factors possibly affecting their practice.

## Methods

### Study design

A descriptive cross-sectional and non-participant structured observational design was utilized in the intensive care units (ICUs) of four hospitals in Hodeida city, Yemen. The data were collected using a 25-items observational checklist in the period from May to August 2019. This design is more suitable to measure aspects or events related to human behaviors [18].

### Setting and study participants

The present study was conducted in the intensive care units (ICUs) of one public (Al-Thawora) and three private (Al-Amal, Al-Aqsa and Al-Rasheed) hospitals where the ETS procedure was performed for mechanically ventilated patients in Hodeida city which is considered the most populated after Sana’a (the capital of Yemen). Al-Thawora hospital is considered the largest hospital in Hodeida that has two ICUs (general and cardiac) with 30-bed capacity. The General ICU consists of 20 beds and three mechanical ventilators (MVs) and the Cardiac ICU contains 10 beds and two MVs. The private hospitals contain general ICUs that have a varied number of beds from 10 to 20. CCNs who were directly providing ETS procedure for the mechanically ventilated patients and who had at least 1 year of experience in the ICUs were involved in the present study. CCNs working in ICUs, who do not provide ETS for mechanically ventilated patients, were excluded. Eighty-three of CCNs were working in the intended ICUs, three of them were excluded as they were in a part during the period of study. The actual participants in our study were eighty of CCNs.

### Tool of the study

The data were collected using a CCNs’ socio-demographic sheet and a 25-items observational checklist that were developed by the researchers after review the previous recommendations and studies [4, 17, 19]. The items were grouped to four parts according to the steps of ETS procedure including 6-items related to practices related to prior ETS, 5-Items related to infection control, 6-items related to practices during ETS, and 8-items related to practices post ETS. The score of each item was rated as either 1 (done) or 0 (not done) where the total scores for the checklist ranged from 0 to 25.

In addition, CCNs’ socio-demographic sheet consists of information related to CCNs including gender, age, level of education, name of the hospital, years of work experience, years of professional and ICU experience, being trained or educated for the prevention of ETS. To minimize the Hawthorne effect, the researchers collected data during two stages. Stage one implemented by one researcher where he visited the participants to complete the demographic data and the informed consent during May 2019. The second stage was implemented by researchers’ assistances, who were selected from each hospital and trained and supervised by the main researcher, to observe and evaluate the ETS practice during the period between July–August 2019. The participants were observed for their ETS practice on one shift a day either in morning or evening.

### Validity and reliability

The observational checklist was reviewed by six nursing professionals with experience in ICUs. Their comments for improving the words’ accuracy and the items’ understandability were considered. Based on their suggestions, we adjusted a few words but nothing was added or removed. A pilot study was conducted to assess the checklist’s inter-rater reliability. Eight ICU nurses from three different ICUs in Hodeida were evaluated independently by one researcher and a senior nurse. The raters repeated the evaluation 2 weeks later. The ratings of the two observers were then compared, and their levels of agreement were checked; reliability was found to be acceptable for all checklist’s items.

### Ethical approval

The study was ethically approved by the Ethics Committees of the Faculty of Medicine and Health Sciences, Hodeida University (Ref. No. 282/2019). Permission was taken from the hospitals’ administrations and units’ managers where the study was conducted. Written consent illustrating the purpose, risks and benefits of the study, was obtained from all participants. Participants were assured that their participation in this study is voluntary, and they are free to withdraw at any time. The participants were assured that every effort would be made to protect their anonymity and that only aggregated data would be communicated. Confidentiality of participants was maintained.

### Statistical analysis

The Statistical Packages for Social Sciences (SPSS), version 21.0 (IBM Corp., Armonk, NY, USA) was used for processing and analysis of the data. Descriptive statistics such as frequencies, percentages and means were used to describe the participants’ characteristics and their items of practice. Statistics including frequency ratings, percentage, mean and standard deviations were computed for nominal data and presented in tables.

Each checklist item was scored as 1-point for a correct practice and a 0 for an incorrect one, then the average of score was converted to percentages. The quality of ETS practice was classified into three groups according to the sum of the percentages; undesirable, moderate, and desirable (< 50, 50–75, and 76–100, respectively) [19]. Mann—Whitney *U*, Kruskal—Wallis and Chi-Square tests were used to determine the relationship between demographic characteristics and participants’ practice scores. The significance level for all tests was set at *p* < .05.

## Results

Table 1 illustrates the socio-demographic and occupational characteristics of CCNs. More than a half of the participants were female and have diploma degree (52.5 and 55%, respectively) and the mean age of the participants was 29.2 ± 5.5. The majority of participants (51.2, 66.2, and 57.5%, respectively) had experience less than 5 years, had not trained, and did not receive information regarding the endotracheal suctioning.

**Table 1:** Demographic and occupational characteristics of CCNs (*N* = 80)

Factor	Nurses (*n* = 80) No. (%)
Gender	
Male	38 (47.5)
Female	42 (52.5)
Age (Years)	
Mean ± SD	29.2 ± 5.5
< 26	19 (23.8)
26–30	36 (45)
> 30	25 (31.2)
Education level	
Diploma	44 (55)
Bachelor	36 (45)
Years of experience	
< 5	41 (51.2)
≥ 5	39 (48.8)
Training in ICUs	
Yes	27 (33.8)
No	53 (66.2)
Received Information	
Yes	34 (42.5)
No	46 (57.5)

Values are mean ± SD or n (%)

*ICU* Intensive Care Unit

Table 2 illustrates the CCNs’ practices prior to suctioning. The majority of the CCNs did not auscultate the patients’ chest before suction and did not explain the procedure to their patients (70 and 80%, respectively). About two thirds (68.8 and 65%, respectively) of the patients were put on suitable position and sodium chloride instillation were applied of the endotracheal tube. More than a half (57.7%) chose the correct diameter of suction catheter ≤ Half of the internal diameter of ETT. Regarding the CCNs’ practices during suctioning, more than a half were observed passing the catheter more than 2 times and the length of time suction applied to airway more than 15 seconds in most of their practice (56.2 and 58.8%, respectively) while all of the them did not adjust the suction pressure. The suctions were applied only during withdrawal the catheters from the airways in the most practices (66.2%) while “humidification by passing saline through the suction catheter” was the lowest performed practices among the CCNs. Regarding CCNs’ practices post suctioning. Patient were reconnected to oxygen in the period of more than 10 seconds post suctioning in most cases (60%). The items of “Postsuctioning hyper-oxygenation/hyperinflation”, “Giving oral care”, “Used catheter and gloves are disposed of in a manner that prevents contamination from secretions” were the most practices of the CCNs while the items of “Auscultating the patient’s chest after ETS”, “Patient reassured”, were the lowest practices among the CCNs.

**Table 2:** CCNs’ practices in relation to evidence prior to, during and post ETS (*N* = 80)

Practice period	Item	Variable	no (%)
**Prior ETS**	1. Auscultating the patient’s chest before ETS?	Yes	24 (30%)
		No	56 (70%)
	2. Explaining the procedure to the patient?	Yes	16 (20%)
		No	64 (80%)
	3. Putting the patient in a suitable position?	Yes	55 (68.8%)
		No	25 (31.2%)
	4. Pre-suctioning hyper-oxygenation/ hyperinflation	Given	24 (30%)
		Not given	56 (70%)
	5. NS instillation	Yes	52 (65%)
		No	28 (35%)
	6. Diameter of suction catheter/ETT	>Half of ETT	34 (42.5%)
		≤ Half of ETT	46 (57.5%)
**During ETS**	7. Number of suction passes	> 2 times	45 (56.2%)
		≤ 2 times	35 (43.8%)
	8. Length of total suction time applied to airway	More than 15 seconds	46 (58.8%)
		Less than 15 seconds	34 (41.2%)
	9. Adjusting the correct aspirator pressure level of suction pressure	< 80 / > 150 mmHg	80 (100%)
		80–150 mmHg	0 (0%)
	10. Time of suction applied	during insertion	27 (33.8%)
		during withdrawal	53 (66.2%)
	11. Humidification by passing saline through the suction catheter	Yes	24 (30%)
		No	56 (70%)
	12. Resting of the patient for 30—60 s in between consecutive suctions	Yes	46 (57.5%)
		No	34 (42.5%)
**Post ETS**	13. Patient reconnected to oxygen post suction	> 10 seconds	48 (60%)
		within 10 seconds	32 (40%)
	14. Postsuctioning hyper-oxygenation/hyperinflation	Given	21 (26.2%)
		Not given	59 (73.8%)
	15. Giving oral care	Yes	44 (55%)
		No	36 (45%)
	16. Auscultating the patient’s chest after ETS?	Yes	33 (41.2%)
		No	47 (58.8%)
	17. Patient reassured	Yes	28 (35%)
		No	52 (65%)
	18. Monitoring vital signs	Yes	41 (51.2%)
		No	39 (48.8%)
	19. Hands washed post-suctioning	Yes	45 (56.2%)
		No	35 (43.8%)
	20. Appropriate removing of used items	Yes	62 (77.5%)
		No	18 (22.5%)

Table 3 shows the Infection control measures for the CCNs’ practices. The majority (67.5%) of the CCNs’ didn’t do the hand washing prior to suctioning. Most of the CCNs worn the gloves and did not wear apron or face mask and all of them didn’t wear the goggles.

**Table 3:** Infection control measures for the CCNs’ practices (*N* = 80)

#	Item	Variable	n (%)
1	Hands are washed prior to suctioning	Yes	26 (32.5%)
		No	54 (67.5%)
2	Gloves are worn	Yes	68 (85%)
		No	12 (15%)
3	Apron is worn	Yes	12 (15%)
		No	68 85%)
4	Face mask is worn	Yes	27 (33.8%)
		No	53 (66.2%)
5	Goggles are worn	Yes	0 (0%)
		No	80 (100%)

Table 4 illustrates the overall scores of CCNs’ practice. More than a half (55%) of CCNs scored undesirable (less than 50%), while less than a half (45%) scored moderate (50–75%), and none of them scored at the desirable level (more than 75%).

**Table 4:** Overall scores of CCNs’ practice

Level	***n*** = 80	Percent
< 50%	44	55%
50–75%	36	45%
> 75%	–	–

Table 5 illustrates the relationship between the CCNs’ sociodemographic characteristics and their practice. There was no relationship between their practice and the items of “gender”, “education level”, “their experience in ICU”, and “age”, (*p* = 0.053, 0.090, 0.410, and 0.739, respectively) while there was a relationship between their practice and items of “training on ETS” and “received information of ETT”, (*p* = 0.010 and 0.028, respectively).

**Table 5:** Factors associated with CCNs’ practice to the guideline (*n* = 80)

Factors	Group	N	Mean Rank	Median (IQR)	***p***-value
Gender^a^	Male	38	45.99	13 (11–16)	0.053
	Female	42	35.54	11 (10–13.25)	
Education Level^a^	Diploma	44	36.55	12 (10–13.75)	0.090
	Bachelor	36	45.33	12 (11–16)	
Training on ETS^a^	Yes	27	49.81	14 (11–16)	0.**010**
	No	53	35.75	12 (10–13)	
Received information for ETS^a^	Yes	34	47.09	13 (10.75–16)	**0.028**
	No	46	35.63	12 (10–13)	
Experience in ICU (years)	< 5	41	(38.43)	12 (10–14)	0.410
	≥ 5	39	42.68	n (10–15)	
Age category (years)^b^	< 26	19	42.95	12 (10–14)	0.739
	26–30	36	38.33	11.5 (10–14)	
	> 30	25	41.76	13 (10–14.5)	

^a^Mann-Whitney test

^b^Kruskal-Wallis test was conducted at α = 0.05

## Discussion

The practice of ETS by CCNs is one of the frequently applied procedures for the management and safety of critically ill patients in ICUs. Several studies investigated the CNNs’ practice regarding ETS [4, 13, 19]. To our knowledge, no studies have been published about the practice of ETS among CCNs in Yemen. In the present study, the adherence level of CCNs to ETS practice guidelines was either undesirable (by 55%) or moderate (by 45%) when performing the procedure for critically ill patients, with none of them showing desirable adherence to guidelines. This finding is consistent with that reported from Sudan [9], where fair (scored < 50%) and poor (scored 50–70%) levels of practice were observed for 76.6 and 23.4% of CCNs, respectively, while none of them showed a good level of practice (scored > 70%). Likewise, consistent findings have been reported from Finland [8], China [13], and Pakistan [20]. Although 59.7% of Turkish CCNs have been recently found to have a very good level of knowledge of ETS guidelines, only 18.1% of them showed a good level of practice [4]. On the other hand, undesired levels (scored *<* 50%) of knowledge and practice of suctioning were observed among 80.6 and 85.7% of Tanzanian CCNs, respectively [19]. Generally, the gap between the CCNs’ knowledge can be bridged by providing them with regular courses and training based on evidence-based clinical guidelines [4, 13, 19, 21]. A national survey in Sweden showed that poor staff adherence to mechanical ventilation guidelines could be attributed to several barriers such as lack of training, lack of awareness, resistance to change, and inadequate administrators’ support [22]. The unavailability of ETS guidelines and absence of continuous education and training may ultimately lead to differences in nursing practice [10]. Therefore, Yemeni CCNs need regular courses and training in ETS to gain more knowledge and practice regarding the care of patients with mechanical ventilators. A recent multi-centre survey revealed low knowledge among Yemeni health care workers, including CCNs, regarding the prevention of ventilator-associated pneumonia [23]. Because the present study only assessed CCNs’ adherence to ETS practice guidelines, further studies are recommended to investigate CNNs’ level of knowledge about ETS guidelines.

Evidence-based studies recommend the importance of performing respiratory auscultation before ETS to determine whether suctioning is needed when secretions are present, which should not be performed routinely [14, 16, 24–26]. However, our study showed that the majority of CCNs (70%) did not perform auscultation before ETT suctioning. This finding is consistent with that reported in other studies [4, 9, 19], where the majority or none of the CCNs performed auscultation before ETT suctioning [4, 7, 9, 19]. A possible explanation is that CCNs might depend on their experience rather than clinical evidence to assess the need for suctioning [4, 17]. Accordingly, such practices contradict the evidence that suctioning should be indicated and performed only when necessary [14, 16, 25].

Exposure of patients to ETS is often associated with anxiety and discomfort [6], and CCNs play a key role in alleviating this anxiety and promoting patient understanding and compliance by providing patients with clear information about the need for ETT suctioning and the consequences of not performing it when required [24]. In the present study, the proportion of CCNs (80%) who did not explain the suctioning procedure to patients is higher than that reported for CNNs in Sudan (26.7%) [9] and Ethiopia (51%) [7]. On the other hand, a lower proportion (2.8%) of Turkish CCNs explained the procedure to their patients [4]. In the present study, approximately 30% of CCNs performed pre-hyper-oxygenation/hyperinflation, which is slightly higher than that (20%) reported for Turkish CCNs [4]. On the contrary, it is lower than that reported in other recent studies [7, 13, 21]. It is noteworthy that pre-hyper-oxygenation (oxygen at 100% for 30 to 60 seconds prior to the suctioning event) strategy can help reduce some ETT suctioning complications, such as hypoxemia, cardiac arrhythmia, cardiac and/or respiratory arrest, and even death [14, 16]. The lack of written clinical guidelines and training programs in the surveyed ICUs could be a major reason for such practice, where CCNs may be unaware of its complications.

Approximately two-thirds of the CCNs in this study used normal saline (NS) prior to suctioning in spite of the lack of evidence about the benefit of such practice. This finding is consistent with that reported in a recent study compiling surveys from 20 countries [21]. A recent study in Ethiopia showed that only less than one-third of CCNs were aware of the contraindication of using NS during ETS, while more than two-thirds used it during the procedure [7]. On the other hand, another study showed that all Canadian CCNs used NS prior to suctioning [27]. On the contrary, only one CCN was found not to use NS prior to suctioning in Turkey [4]. It is worth mentioning that there is evidence that using NS during suctioning can lead to adverse consequences such as the increased risk of infection, reduced oxygen saturation, patient discomfort, and increased amount of secretions [15, 28, 29]. A possible explanation for this practice is the misconception of CCNs that NS humidifies and clears respiratory secretions [27].

The use of a small suction catheter with external diameters not exceeding one-half of the internal diameter of the ETT is recommended to allow air entry into the lungs during suctioning, consequently preventing the development of excessive negative pressure and potential atelectasis [16, 25, 30]. In the present study, the majority (57.5%) of the used catheter sizes were appropriate (less than half the internal diameter of ETT). This finding agrees with that reported for CCNs in China [13]. In contrast to the present finding, a study in Turkey found that almost all CCNs had used catheters of the correct diameter size [4]. However, more than half of their participants chose the catheter size based on visual recognition or color. Likewise, a study in Tanzania found that the majority of CCNs (86.4%) were unaware of determining the correct size of endotracheal catheters for suctioning, even though more than half (57.1%) of them selected the appropriate size during their practice [19]. A possible reason for selecting large catheter sizes could be the CCNs’ perception that large sizes could facilitate the removal of thick secretions. However, large catheters can lead to hypoxemia and trauma.

ETS is an invasive procedure that requires adherence to aseptic technique and infection control measures to prevent nosocomial infections due to contamination of the lower respiratory tract [13]. Critical steps of the aseptic technique include handwashing and wearing gloves. In the present study, handwashing and wearing gloves were practiced by 32.5 and 85% of CCNs, respectively. Similarly, a low proportion (12.5, 47%) was reported for CCNs in Turkey [4]. In contrast, a study in China showed that two-thirds of CCNs wore gloves and washed their hands before ETS [13]. Despite wearing gloves by all CCNs in the present study, the use of non-sterile gloves by them is inconsistent with the Clinical Practice Guidelines of the American Association for Respiratory Care (AARC) in 2022 about wearing sterile gloves before ETTs [14]. However, the surveyed ICUs did not provide them with sterile gloves, perhaps due to their higher cost. The CCNs’ adherence to handwashing in the surveyed ICUs could be attributed to several factors such as their limited time, understaffing, and overcrowding [31]. Regarding wearing aprons and goggles, a low proportion (12%) of CCNs wore aprons while none wore goggles, which is in line with a study in Turkey [4]. Lack of equipment, inadequate motivation to prevent infection could be the reasons for non-adherence of CCNs to these practices.

The findings of this study showed no association between gender, age, education level, and length of experience of CCNs in the ICUs and their practice during performance ETS procedures. Similarly, recent studies in China and Tanzania found no association [13, 19]. Because more than half of CCNs (55%) in the present study held diploma degrees, the administrators of the surveyed hospitals need to exert further efforts to upgrade the education level of CCNs in the ICUs. On the other hand, training on ETS and receiving information about ETS significantly affected the CCNs’ practice. This finding highlights the importance of continuous education and training of CCNs in improving their practice during ETS of critically ill patients undergoing mechanical ventilation.

### Strengths and limitations of the study

This study was the first to assess the adherence of CCNs to the guidelines of ETS in Hodeida hospitals, Yemen. Its findings can provide recommendations for improving ETS practices and raise awareness of CCNs and hospital administrators about the guidelines of ETS**.** However, the present study has a number of limitations. First, the study assessed CCNs’ practice of ETS, but not their knowledge. Accordingly, the gap between knowledge and practice in this issue could not be identified. Second, like many other observational studies, the Hawthorne effect may have potentially changed the CCNs’ practice due to the CCNs’ feeling of being directly observed. However, to minimize this effect, data were collected over two separate periods. Third, the sampling of CCNs from ICUs in Hodeida city may limit the generalizability of the findings of the study.

## Conclusion and recommendations

This study provides important insights into the practices of Yemeni CCNs when performing ETS procedures. Most of CCNs at the ICUs of Hodeida hospitals do not adhere to evidence-based practice guidelines when performing ETS procedures, possibly resulting in numerous adverse effects and complications for patients. CCNs receiving information and practice programs show better ETS practice than their counterparts do. Therefore, it is necessary to provide the nursing staff with clear guidelines, continuous education and monitoring to improve their practices. Educational nursing institutions should incorporate evidence-based ETS practices into their curricula. Health care administrators should pay more attention to providing up-to-date guidelines, continuous training and monitoring for CCNs besides providing the ICUs with the equipment and devices necessary for performing ETS. Further studies are required to investigate the gap between knowledge and practice as well as the barriers and facilitators for implementing ETS guidelines.

## Data Availability

The datasets used and/or analyzed during the current study are available from the corresponding author on reasonable request.

## Abbreviations

ETT: Endotracheal tube
ETS: Endotracheal suctioning
CCNs: Critical care nurses
ICUs: Intensive care units
SPSS: Statistical Packages for Social Sciences
